# Peer Review of “Emergence of the First Strains of SARS-CoV-2 Lineage B.1.1.7 in Romania: Genomic Analysis”

**DOI:** 10.2196/32299

**Published:** 2021-08-13

**Authors:** Lei Guo

**Affiliations:** 1 Washington University School of Medicine in St. Louis St Louis, MO United States

**Keywords:** infectious disease, COVID-19, strain, virus, Romania, transmission, spread, mutation, impact, case study, genome, sequencing, genetics, epidemiology, variant, virology, lineage

*This is a peer-review report submitted for the paper “Emergence of the First Strains of SARS-CoV-2 Lineage B.1.1.7 in Romania: Genomic Analysis.”*

## Round 1 Review

### General Comments

First of all, the authors presented an important work about the new UK variant of COVID in Romania [1]. I have the following questions.

### Specific Comments

#### Major Comments

1. In the Methods section, the authors mentioned that “Twenty samples, collected from patients in the cities of Cluj, Craiova and Suceava counties from Romania were selected for analysis, including patients with possible contacts with UK infected individuals.” In the Introduction section, the authors also described the first few possible UK variant cases in Romania.
  Are these 20 cases sequenced by authors related to those cases mentioned in the Introduction? If not, can authors provide some details about the subjects' past travel history? For example, did they stay in UK for more than 2 weeks before they traveled to Romania? And when were these samples collected? The timeline is important to understand how the disease spread and whether they are the first strains of B.1.1.7 in Romania.
2. The authors claimed that “the Romanian strains bearing the particular ORF8 mutations described above clearly originated in the UK, which is also supported by the fact that the patient from Suceava county arrived in Romania from the UK.” I have a similar question about the travel details of the patient as well as the timeline.
3. From a public health standpoint, how did the authors deal with the “news” of the new variant? Was there any communication with local officials or support for contact tracing?
4. In the Discussion section, the authors described that “Many European countries, including Romania, lag in genomic sequencing”. Can the authors provide more details about why Romania lags in genomic sequencing for COVID? For example, cost, equipment, access to labs/institutes. This can help readers and other researchers to understand the issue.
